# Hepatitis B Doubly Spliced Protein (HBDSP) Promotes Epithelial‐Mesenchymal Transition, Migration, and Invasion via SP1/ETS1‐Dependent YAP Activation in Hepatoma Cells

**DOI:** 10.1002/jmv.71046

**Published:** 2026-07-09

**Authors:** Xiazhen Xu, Yibin Peng, Yuxin Ping, Tiantian Shao, Qiong Wu, Lu Zhang, Xu Lin, Changxi Yu, Wannan Chen

**Affiliations:** ^1^ Key Laboratory of Gastrointestinal Cancer (Fujian Medical University), Ministry of Education, School of Basic Medical Sciences Fujian Medical University Fuzhou China; ^2^ Fujian Key Laboratory of Tumor Microbiology, Department of Medical Microbiology, School of Basic Medical Sciences Fujian Medical University Fuzhou China

**Keywords:** EMT, HBDSP, hepatitis B virus, invasion, RNA splicing, YAP

## Abstract

Chronic hepatitis B virus (HBV) infection remains a significant global health burden. Although the canonical HBV proteins are well‐studied, the pathological roles of HBV spliced variants and their encoded proteins are poorly understood. In this study, we investigate the cancer‐promoting potential of hepatitis B doubly spliced protein (HBDSP), encoded by the 2.2 kb doubly spliced variant of HBV, and its underlying mechanism in hepatocellular carcinoma (HCC). In vitro functional assays demonstrated that HBDSP induces epithelial‐mesenchymal transition (EMT) and enhances migration and invasion in HepG2 and Huh7 cells. Mechanistically, cellular and molecular approaches revealed that HBDSP enhanced the nuclear translocation of SP1 and ETS1, thereby facilitating their binding to the yes‐associated protein (*YAP*) promoter and activating *YAP* transcription. Inhibition of YAP using either the pharmacological inhibitor Verteporfin or *YAP*‐specific siRNA effectively abolished HBDSP‐induced malignant phenotypes, confirming the essential role of YAP in this process. Notably, these effects were consistently validated in both HBV‐replicating HepG2.2.15 cells and HBV‐infected HepG2‐NTCP cells, further reinforcing the pathological relevance of HBDSP in HBV‐driven hepatocarcinogenesis. Taken together, we identify HBDSP as a novel viral effector that facilitates EMT, migration, and invasion in hepatoma cells via transcriptional activation of *YAP* in an SP1‐ and ETS1‐dependent manner. These findings suggest that HBDSP‐mediated activation of YAP may play a role in HBV‐driven HCC progression and could be explored further as a potential therapeutic axis, particularly in contexts where spliced HBV variants are prevalent.

## 1 Introduction

Chronic hepatitis B virus (HBV) infection is a major cause of chronic hepatitis B (CHB), hepatic cirrhosis, and hepatocellular carcinoma (HCC), contributing to nearly 1 million deaths annually [1]. In 2022, approximately 254 million people were affected by CHB worldwide [2]. Although mass vaccination programs have reduced the incidence of new infections, the high prevalence of chronic carriers and the inability of current therapies to eliminate the virus continue to drive substantial HBV‐related morbidity and mortality, presenting a persistent global public health challenge [1]. This underscores the urgent need for novel therapeutic strategies. HBV is a small DNA virus with a 3.2 kb genome that produces five major transcripts and encodes multiple functional proteins essential for virus replication and pathogenesis [3]. Alternative splicing of the 3.5 kb pre‐genomic RNA (pgRNA) in HBV occurs at particular donor and acceptor sites, generating multiple spliced variants that encode unique viral proteins [4]. These spliced variants have been implicated in viral replication, immune evasion, and disease progression, although their precise roles remain incompletely understood.

To date, at least 20 distinct spliced variants of HBV (designated sp1‐sp20), generated from pgRNA through alternative splicing, have been identified. These variants exhibit differential expression levels and may play diverse roles during the viral infection process and in virus–host interactions [5]. It has been shown that spliced variants of HBV are commonly observed in more than 80% of patients with chronic HBV infection, and the levels of spliced HBV (spHBV) are significantly higher in the sera of patients with HCC compared to those without HCC, suggesting a potential link between elevated spHBV levels and increased risk of HCC development [6]. Among these spliced variants, several (e.g., sp1, sp3, sp7, sp9, sp10, and sp13) contain novel open reading frames (ORFs) formed via splice junctions and frame shifts, potentially encoding truncated or fusion proteins with distinct biological activities, which may contribute to the development of HCC [7]. However, the functions of only four such spliced proteins have been partially elucidated up to now. The hepatitis B spliced protein (HBSP), encoded by the sp1 variant, is a 93‐amino‐acid fusion protein that initiates at the polymerase start codon and contains the N‐terminal 47 residues of the polymerase, followed by 46 residues generated from a novel sequence resulting from a frameshift downstream of the splice junction [8]. HBSP has been reported to promote the migration and invasion of HCC cells and induce hepatoma cell apoptosis [8, 9, 10]. Another sp1‐encoded protein, p21.5, has been shown to inhibit nucleocapsid formation and consequently suppress HBV replication [11]. In contrast, the sp1‐encoded core truncated protein HBc^‐Cys^ was recently found to promote nucleocapsid formation and be essential for HBV infection [12].

The other spliced protein, hepatitis B doubly spliced protein (HBDSP), is encoded by the sp7 variant, which undergoes two splicing events (splicing from nucleotides 2447 to 2935 and 3018 to 489). In this study, the 2.2 kb doubly spliced HBV genome encoding HBDSP, designated FD18‐3 (GenBank accession no. FJ151414.1), was originally isolated from a patient infected with genotype C HBV. HBDSP is a 139‐amino‐acid protein that also initiates at the polymerase start codon. The 47 amino acids in the N‐terminal region are homologous to the N‐terminus of the polymerase, residues Leu48–Gln75 correspond to the preS1 region and represent the critical transactivation domain, and the 64 residues in the C‐terminal segment are derived from a novel frameshifted sequence not homologous to any known HBV ORF [13]. Functionally, sp7 has been shown to promote in vitro replication of wild‐type HBV (wtHBV), suggesting a potential role in persistent infection in HCC patients [14]. Our previous studies demonstrated that HBDSP exhibits transactivational activity on multiple host oncogenes and viral gene promoters through interaction with AP‐1 and C/EBP response elements [13]. Moreover, we previously detected HBDSP expression using an anti‐HBDSP antibody in Huh7 cells transiently transfected with FD18‐3 [13], as well as in HBV‐expressing cell lines HepG2.2.15, HepAD38, and HepG2‐NTCP [15]. Additionally, HBDSP transactivates *p53* via ETS1, GATA2, and YY1, thereby inducing apoptosis in hepatoma cells and facilitating the release of viral antigen and HBV DNA [15]. Beyond apoptosis, we hypothesize that HBDSP may influence additional aspects of hepatoma cell behavior that could potentially contribute to HCC progression, although this remains to be investigated.

HCC is a highly aggressive malignancy with a strong propensity for invasion, metastasis, and frequent recurrence [16, 17]. These malignant features are driven by complex molecular mechanisms, among which epithelial‐mesenchymal transition (EMT) is widely recognized as a critical prerequisite for the invasive and metastatic behavior during HCC progression [18, 19]. The occurrence of EMT is regulated by multiple signaling cascades, which are frequently deregulated in cancers, thereby contributing to aberrant EMT [20].

In this study, we aimed to investigate the potential roles and underlying mechanisms of HBDSP in hepatoma cell metastasis‐related behaviors. Our results showed that HBDSP induced EMT and enhanced cell migration and invasion in HepG2 and Huh7 cells. Screening of EMT‐related signaling pathways demonstrated that HBDSP upregulated the expression of yes‐associated protein (YAP), a critical effector of the Hippo pathway, without altering its phosphorylation status. YAP activation occurs early in hepatocarcinogenesis and is associated with more aggressive HCC subtypes [21, 22, 23]. Our study demonstrated that HBDSP facilitated the nuclear translocation of the transcription factors SP1 and ETS1, thereby enhancing their binding to the *YAP* promoter and driving its transcriptional activation, hence triggering its downstream target genes. Using Verteporfin, a YAP inhibitor, and *YAP*‐specific small interfering RNAs (siRNAs), we confirmed that the EMT‐, migration‐, and invasion‐promoting effects of HBDSP were YAP‐dependent. Furthermore, in HepG2.2.15 and HepG2‐NTCP cells harboring the HBV genome, HBDSP also facilitated EMT, migration, and invasion by regulating YAP. These findings suggest that HBV spliced protein HBDSP may serve as a novel viral factor linking HBV infection to cancer metastasis via modulation of YAP.

## 2 Materials and Methods

### 2.1 Cell Lines and Culture

The human hepatoma cell line HepG2 was procured from the American Type Culture Collection (ATCC; Manassas, VA, USA) and cultured in Minimum Essential Medium (MEM; Thermo Fisher Scientific, Waltham, MA, USA) containing 10% fetal bovine serum (FBS; PAN‐Biotech, Aidenbach, Bavaria, Germany). The human hepatocellular carcinoma cell line Huh7 was obtained from the China Center for Type Culture Collection (CCTCC; Wuhan, China) and maintained in Dulbecco's Modified Eagle Medium (DMEM; Thermo Fisher Scientific) supplemented with 10% FBS. The HBV‐expressing human hepatoma cell line HepG2.2.15, purchased from the CCTCC, was cultured in MEM supplemented with 10% FBS and 380 µg/mL G418 (Sigma‐Aldrich, St. Louis, MO, USA) to maintain stable expression of the integrated dimeric HBV genome. The HepAD38 and HepG2‐NTCP cell lines were kindly provided by Quan Yuan (Xiamen University) and maintained in DMEM supplemented with 10% FBS. In HepAD38 cells, supplementation with tetracycline (Tet; 6 μg/mL; MCE, Monmouth Junction, NJ, USA) suppressed the generation and release of HBV particles, whereas removal of Tet from the culture medium restored HBV production and secretion. All of these cells were grown at 37°C in a humidified incubator under 5% CO_2_.

### 2.2 HBV Production and Infection

HBV particles were obtained from the cell culture supernatants of HepAD38. In brief, supernatants were carefully collected and mixed with polyethyleneglycol 8000 (PEG8000; 8%; Sigma‐Aldrich) under gentle rotation at 4°C for 12–16 h to precipitate viral particles. The samples were then subjected to centrifugation at 10 000*g* for 30 min at 4°C. The resulting viral pellet was resuspended in DMEM at a volume corresponding to 1% of the initial supernatant.

For infection assays, HepG2‐NTCP cells were plated onto collagen‐Ⅰ‐coated 35‐mm dishes and cultured for 72 h in DMEM supplemented with 10% FBS and doxycycline (Dox; 1 mg/mL; TaKaRa, Kusatsu, Shiga, Japan). Cells were subsequently exposed to HBV at a multiplicity of infection (MOI) of 200 in the presence of 8% PEG 8000 and 1.5% dimethyl sulfoxide (DMSO; Sigma‐Aldrich).

### 2.3 Plasmid Construction

The plasmids phouge‐HBDSP, phouge‐HBDSPΔ48‐75, pDsRed‐HBDSP, pcDNA3.1‐ETS1, and pAcGFP‐ETS1 were constructed in our laboratory and have been described previously [15]. To construct SP1 (NM_138473.3) expression plasmids (pcDNA3.1‐SP1 and pAcGFP‐SP1), Flag‐tagged SP1 was inserted into the pcDNA3.1/Hygro(+) vector (Invitrogen, Carlsbad, CA, USA) via the *Nhe Ⅰ* and *Kpn Ⅰ* sites (New England BioLabs, Beverly, MA, USA), and GFP‐tagged SP1 was inserted into the pAcGFP1‐Hyg‐N1 plasmid (Clontech, Mountain View, CA, USA) via the same sites.

The coding sequence of *YAP* (NM_001130145.3) was inserted into the pcDNA3.1‐myc‐His(A) vector (Invitrogen) at the *Nhe Ⅰ* and *Kpn Ⅰ* sites to construct the pcDNA3.1‐YAP‐myc recombinant plasmid. The *YAP* promoter region was obtained from the UCSC Genome Browser platform (https://genome.ucsc.edu). A DNA fragment spanning the −2000 to +200 region of the *YAP* promoter was synthesized by General Biosystems (Anhui, China) and cloned into the the *Kpn I* and *Xho I* sites of pGL4.10 luciferase reporter vector (Promega, Madison, WI, USA) to generate the luciferase reporter construct pGL4.10‐YAP‐2000. A series of 5′‐deletion constructs was generated using pGL4.10‐YAP‐2000 as the template, including pGL4.10‐YAP‐1505 (nucleotides −1505 to +200), −1275 (nucleotides −1275 to +200), −1074 (nucleotides −1074 to +200), −846 (nucleotides −846 to +200), −542 (nucleotides −542 to +200), and −259 (nucleotides −259 to +200). These promoter fragments were PCR‐amplified and cloned into the *Kpn I* and *Xho I* sites of the pGL4.10 luciferase reporter vector. The pGL4.10‐YAP‐542 construct (abbreviated as p542) was subsequently used as the template to generate site‐directed mutants targeting the predicted binding sites of the transcription factors SP1, ETS1, NFIC, and TFAP2A. The mutant constructs included pGL4.10‐YAP‐542‐SP1‐mut (abbreviated as p542‐SP1‐mut); pGL4.10‐YAP‐542‐ETS1‐mut1, ‐mut2 (abbreviated as p542‐ETS1‐mut1, ‐mut2); pGL4.10‐YAP‐542‐NFIC‐mut1, ‐mut2 (abbreviated as p542‐NFIC‐mut1, ‐mut2); and pGL4.10‐YAP‐542‐TFAP2A‐mut1, ‐mut2 (abbreviated as p542‐TFAP2A‐mut1, ‐mut2). The sequences of the predicted transcription factor binding sites, including their wild‐type and mutant forms, are detailed in Table S1, and the sequences of the primers used for PCR amplification are provided in Table S2.

### 2.4 Transfections

Transient transfections with siRNAs or plasmids were performed utilizing Lipofectamine 3000 reagent (Invitrogen) following the protocol provided by the manufacturer. siRNAs for specifically targeted knockdown of *YAP*, *SP1*, and *ETS1* were obtained from GenePharma (Shanghai, China). A scrambled siRNA (siNC) served as a negative control. The sequences of all siRNAs used are detailed in Table S3.

### 2.5 Chemicals

Verteporfin was obtained from MCE and solubilized in DMSO. After transient transfection with plasmids for 24 h, cells were treated with 1.5 µM Verteporfin for an additional 24 h to inhibit YAP activity.

### 2.6 RNA Isolation and Real‑Time Quantitative Polymerase Chain Reaction (qPCR)

The TRIzol Reagent (Invitrogen) was used to isolate the total RNA from HepG2 and Huh7 cells. Subsequently, reverse transcription was conducted using the PrimeScript RT reagent Kit with gDNA Eraser (TaKaRa) to generate cDNA from the purified RNA. The TB Green Premix Ex Taq II (TaKaRa) was employed for qPCR with gene‐specific primers (Brogen Biotechnology, Xiamen, China) on a Stratagene Mx3000P system (Agilent Technologies, Santa Clara, CA, USA). GAPDH was employed as the internal reference gene, and the relative mRNA expression levels were determined using the 2^−ΔΔ^ ^Ct^ method. The primer sequences for this process are provided in Table S4.

### 2.7 Western Blot Assay

Total, cytoplasmic, and nuclear proteins were isolated using the Cell Lysis Buffer (Beyotime Biotechnology, Shanghai, China) and the NE‐PER Nuclear and Cytoplasmic Extraction Reagents (Thermo Fisher Scientific), respectively. Protein concentrations were quantified using the BCA Protein Assay kit (Pierce Biotechnology, Rockford, IL, USA). Equal amounts of protein samples were separated by 10% or 12% SDS‐PAGE and transferred onto 0.45 μm polyvinylidene difluoride (PVDF) membranes (Cytiva, Westborough, MA, USA) for immunoblotting. Membranes were blocked with 1% bovine serum albumin (BSA) for 2 h at room temperature and incubated overnight at 4°C with specific primary antibodies. After washing three times with 1×TBST, the membranes were incubated with appropriate secondary antibodies for 1 h at room temperature, followed by three additional washes with 1×TBST. Protein bands were visualized using an enhanced chemiluminescence (ECL) substrate (Amersham, Buckinghamshire, UK) and imaged with an Amersham ImageQuant 800 system (Cytiva, Westborough, MA, USA). Band intensities were quantified using ImageJ software (National Institutes of Health, Bethesda, MD, USA), normalized to the corresponding loading controls, and presented as fold changes relative to the control group. All Western blot data were obtained from three biologically independent experiments, each involving separate transfections. A variety of primary and secondary antibodies were used including anti‐Flag (1:1000) (Sigma‐Aldrich); anti‐GAPDH (1:1000), anti‐Histone H3 (1:1000), anti‐E‐cadherin (1:1000), anti‐N‐cadherin (1:1000), anti‐Vimentin (1:1000), anti‐Snail (1:1000), anti‐Notch1 (1:1000), anti‐Cleaved Notch1 (1:1000), anti‐p44/42 (1:1000), anti‐phospho‐p44/42 (1:1000), anti‐p38 (1:1000), anti‐phospho‐p38 (1:1000), anti‐SAPK/JNK (1:1000), anti‐phospho‐SAPK/JNK (1:1000), anti‐YAP (1:1000), anti‐phospho‐YAP (1:1000), anti‐MST2 (1:1000), anti‐LATS1 (1:1000), anti‐CYR61 (1:1000), anti‐CTGF (1:1000), anti‐SP1 (1:1000), anti‐ETS1(1:1000), anti‐myc (1:1000), horseradish peroxidase (HRP)‐linked anti‐rabbit IgG (1:2000) and HRP‐linked anti‐mouse IgG (1:2000) (Cell Signaling Technology, Danvers, MA, USA).

### 2.8 Immunofluorescence Staining (IF) Assay

HepG2 and Huh7 cells were plated in 35 mm confocal dishes and cultured until reaching 60%–70% confluence. Cells were transiently transfected with either the phouge‐HBDSP plasmid or the corresponding control. After transfection, the cells were washed thrice with cold 1×PBS, then fixed in 4% paraformaldehyde for 15 min. After fixation, the cells were rinsed again with 1×PBS, permeabilized, and blocked using 0.1% Triton X‐100 and 5% goat serum (Beyotime Biotechnology) in 1×TBS for 90 min. The cells were subsequently incubated with primary antibodies against E‐cadherin (1:100) or Vimentin (1:200) overnight at 4°C. After intensive washing with 1 × TBS supplemented with 0.1% Triton X‐100, the cells were incubated with Goat anti‐Rabbit IgG (H + L) Cross‐Adsorbed Secondary Antibody, DyLight 594 (Invitrogen) or CoraLite 488‐conjugated Goat Anti‐Rabbit IgG (H + L) (Proteintech, Wuhan, China) for 90 min in the dark. Finally, nuclear staining was conducted with DAPI (Beyotime Biotechnology), and fluorescent images were captured using a Leica TCS SP5 confocal microscope (Leica Microsystems, Teaneck, NJ, Germany).

### 2.9 Wound‐Healing Assay

Wound‐healing assays were conducted to assess the migratory abilities of HepG2, Huh7, HepG2.2.15, and HBV‐infected HepG2‐NTCP cells. Briefly, cells were transfected with the phouge‐HBDSP plasmid alone, co‐transfected with the phouge‐HBDSP plasmid and siRNAs, or transfected with the phouge‐HBDSP plasmid followed by treatment with Verteporfin for the specified time periods. Subsequently, the cells were trypsinized, resuspended in fresh medium, and seeded into 35 mm culture dishes containing 2 mL of serum‐free medium. After 12 h incubation, allowing near‐confluent monolayers to form, a straight scratch was created on the cell monolayer with a sterile pipette tip. Subsequently, cells were rinsed three times with 1×PBS to remove cellular debris. Photographs of the scratch wound were taken at 0, 24, and 48 h with an inverted fluorescence microscope (ZEISS) at 50× magnification. The widths of the scratch gaps at each time point were recorded to assess the extent of cell migration.

### 2.10 Matrigel Invasion Assay

The invasive abilities of HepG2, Huh7, HepG2.2.15, and HBV‐infected HepG2‐NTCP cells were assessed using the Corning BioCoat Matrigel Invasion Chambers (Corning, Corning, NY, USA). Briefly, cells were either transfected with the phouge‐HBDSP plasmid alone, co‐transfected with the phouge‐HBDSP plasmid and siRNAs, or transfected with the phouge‐HBDSP plasmid followed by Verteporfin treatment for specified durations. Subsequently, cells were trypsinized and resuspended in fresh serum‐free medium. A total of 5–10 × 10^4^ cells in 500 μL of serum‐free medium was added to the upper Matrigel‐coated chamber, while 800 μL of medium containing 20% FBS was placed in the lower chamber as a chemoattractant. After incubation for the designated period, non‐invading cells on the top membrane surface were carefully wiped away using a cotton swab. Invaded cells on the underside of the membrane were rinsed three times with 1×PBS, fixed with 4% paraformaldehyde for 30 min, and then stained with crystal violet for 20 min. After discarding the excess dye and rinsing the membranes, invasive cells attached to the bottom surface were imaged and counted under an inverted fluorescence microscope (ZEISS) at 200× magnification. Cell numbers were recorded from six random fields.

### 2.11 Co‐Immunoprecipitation (co‐IP) Assay

Whole‐cell lysates were prepared from transfected HepG2 and Huh7 cells using the Cell Lysis Buffer, and total protein concentrations were subsequently quantified using the BCA Protein Assay kit. For each immunoprecipitation, 1 mg of total protein was incubated overnight at 4°C with either 10 μL of Anti‐Flag Magnetic Agarose Beads (MCE) or 10 μL of Protein A/G Magnetic Beads (MCE) that had been pre‐incubated with anti‐myc or anti‐IgG antibody (Santa Cruz Biotechnology, Dallas, TX, USA). The anti‐IgG antibody was used as a negative control. The lysis buffer was used to wash the beads three times, which were then isolated using the DynaMag‐2 magnetic rack (Thermo Fisher Scientific). Subsequently, immunoprecipitated proteins were eluted in 30 μL of 1×SDS loading buffer by heating, separated on 12% SDS‐PAGE gels, and detected by Western blot using antibodies specific for YAP, Flag‐tag, or myc‐tag.

### 2.12 Luciferase Reporter Assay

Luciferase reporter assays were carried out utilizing the Bright‐Glo Luciferase Assay System (Promega, Madison, WI, USA) following the manufacturer's recommendations. Following three washes with 1× PBS, transfected HepG2 cells were lysed in 1× Glo Lysis Buffer (Promega) for 15 min at room temperature to achieve complete lysis. The lysates were then collected by centrifugation, and the supernatants were determined using the BCA Protein Assay Kit. Equal amounts of protein (30 μg per sample) were adjusted to a final volume of 100 μL with 1× Glo Lysis Buffer and subjected to luciferase activity measurement using an EnSight Multimode Microplate Reader (PerkinElmer, Waltham, MA, USA).

### 2.13 Chromatin Immunoprecipitation (ChIP)

ChIP assays were carried out using a SimpleChIP Enzymatic Chromatin IP Kit (Magnetic Beads) following the supplier's instructions. In brief, HepG2 cells transfected with phouge‐HBDSP or phouge plasmids were cross‐linked with 1% formaldehyde, quenched with 1× Glycine Solution, scraped in cold 1× PBS supplemented with protease inhibitors, and collected by centrifugation. Cells were lysed in a mixture of 1× Buffer A, 1× Buffer B, and 1× ChIP Buffer. The isolated chromatin was then digested with micrococcal nuclease and subsequently sonicated to obtain fragments ranging from 150 to 900 bp. Chromatin fragments containing DNA‐protein complexes were immunoprecipitated using antibodies against SP1, ETS1, or Flag. Negative control immunoprecipitations were performed with anti‐IgG antibodies, and anti‐Histone H3 antibodies were used as a positive control. Protein G magnetic beads were added to the reaction mixtures and incubated for 2 h at 4°C. Immunocomplexes were then eluted with 1× ChIP elution buffer. Cross‐links were reversed using proteinase K and NaCl, and DNA was purified. The immunoprecipitated DNA was then analyzed by PCR or qPCR to amplify regions within the YAP promoter containing SP1 or ETS1 binding sites. The specific primer sequences used are as follow: for PCR, −542/−259nt‐F: 5′‐GGCTTTAAGCTCGCACAGGCGCTCC‐3′; −542/−259nt‐R: 5′‐ GAAGCGTGCCCCGTATTCTGCCCCG‐3′; p5000‐F: 5′‐AAATGTGATGAGTTACGTGA‐3′; p5000‐R: 5′‐TAAACATTTGAAACATACCA‐3′; and for qPCR, −542nt/−259nt‐q‐F: 5′‐GAGCGGAGCGGAAGAACTTC‐3′; −542nt/−259nt‐q‐R: 5′‐GCAAACGATGGGTCCAATCC‐3′.

### 2.14 Electrophoretic Mobility Shift Assay (EMSA)

The LightShift Chemiluminescent EMSA Kit (Thermo Fisher Scientific) was used to perform EMSA as the manufacturer's instructions described. Nuclear extracts were prepared from HepG2 cells following transfection with phouge‐HBDSP or phouge plasmids using the NE‐PER Nuclear and Cytoplasmic Extraction Reagents (Thermo Fisher Scientific). Double‐stranded biotinylated oligonucleotides containing the consensus recognition sequences for SP1 or ETS1 were used as labeled probes. Specific competition was evaluated using unlabeled oligonucleotides (cold probes), and sequence‐specificity was confirmed with site‐directed mutants (cold mutated probes). All oligonucleotides were chemically synthesized in single‐stranded form by SunYa Biotechnology (Fuzhou, China). The oligonucleotide probe sequences are detailed in Table S5. To generate double‐stranded oligonucleotides, equimolar amounts of complementary single‐stranded oligonucleotides were heated to 95°C for 2 min and then allowed to cool slowly to room temperature for annealing. Binding reactions were carried out using biotin‐labeled probes and nuclear extracts in the presence of poly (dI‐dC) and 1× binding buffer. For competition assays, an excess of unlabeled (cold) or mutated (cold mutated) probes was pre‐incubated with the reaction mixtures prior to the addition of the biotin‐labeled probes. Super‐shift assays were conducted using anti‐SP1 or anti‐ETS1 antibodies to confirm the specificity of nuclear protein binding to the corresponding recognition sites. After incubation, DNA‐protein complexes were separated by electrophoresis on 6% native polyacrylamide gels and then transferred onto nylon membranes (Cytiva) for detection. Following UV cross‐linking, the membrane was blocked with 1× blocking buffer and then incubated with streptavidin‐HRP for 15 min. After four washes with 1× washing buffer, the membrane was sequentially incubated with 1× detection buffer and working substrate solution. Finally, imaging was performed using an Amersham ImageQuant 800 system.

### 2.15 Confocal Microscopy

HepG2 cells were seeded onto collagen‐coated confocal plates and co‐transfected with pDsRed‐HBDSP or corresponding empty vector control, together with pAcGFP‐SP1 or pAcGFP‐ETS1 plasmids. After 48 h, the cells were rinsed three times with 1×PBS, fixed in 4% paraformaldehyde for 15 min, and subsequently washed again with 1× PBS. Nuclear staining was carried out using DAPI (Beyotime Biotechnology). Finally, fluorescence images were captured utilizing a Leica TCS SP8 X confocal microscope (Leica Microsystems).

### 2.16 Bioinformatics Analysis

RNA‐seq transcriptomic data from The Cancer Genome Atlas Liver Hepatocellular Carcinoma (TCGA‐LIHC) cohort, including 374 HCC tissues and 50 adjacent normal liver tissues, were downloaded using the R package TCGAbiolinks (v4.5.1). Normalized expression data were used for subsequent analyses. The Gene Expression Omnibus (GEO) dataset GSE83148, comprising liver biopsy samples from 122 CHB patients and 6 controls, was retrieved from the GEO database. Microarray data were processed and annotated using the R packages tinyarray (v2.4.3) and hgu133plus2.db, respectively. Probe IDs were converted to gene symbols according to the corresponding platform annotation files. Differential gene expression between groups was analyzed using the Wilcoxon rank‐sum test. Correlation analyses were performed using Spearman's correlation coefficients.

### 2.17 Statistical Analysis

All statistical analyses and data visualization were performed using GraphPad Prism software, version 9.5.0 (GraphPad Software, La Jolla, CA, USA). Data are presented as the mean ± standard deviation (SD) from three independent experiments. Differences between two groups were analyzed using the Student's *t*‐test, with *p* < 0.05 considered statistically significant.

## 3 Results

### 3.1 HBDSP Induces EMT and Enhances Migration and Invasion

**Figure 1:**
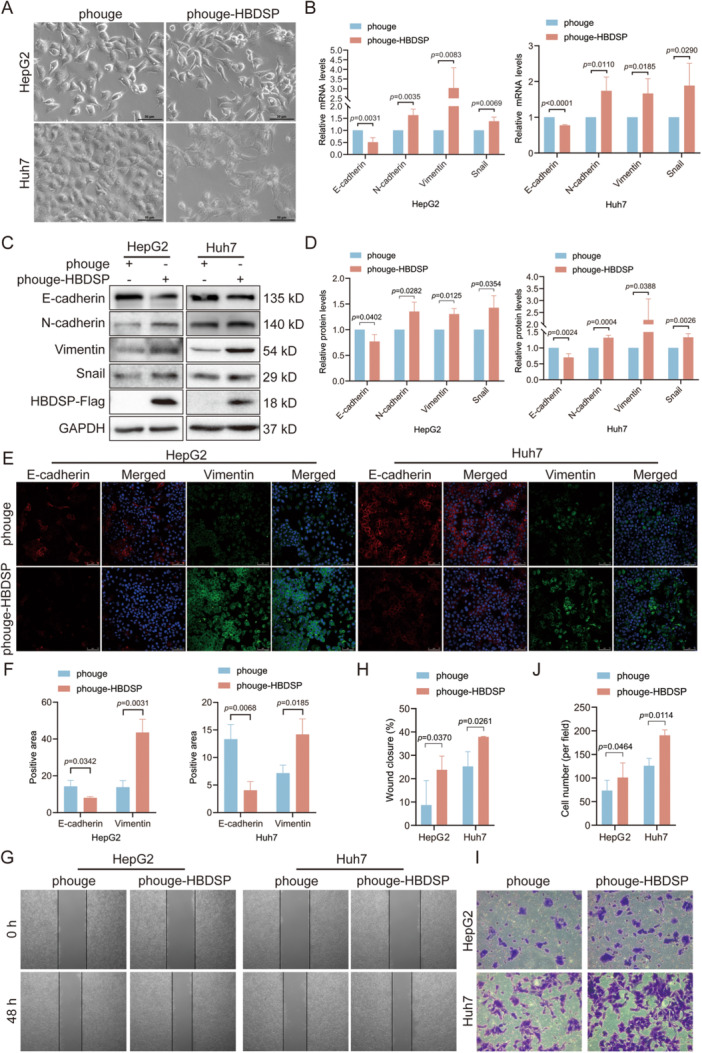
HBDSP overexpression enhances EMT, migration, and invasion in HepG2 and Huh7 cells. (A) Representative cell morphologies were observed under an inverted fluorescence microscope following HBDSP overexpression (scale bars, 50 μm). (B) The expression levels of key EMT markers after HBDSP overexpression were detected by qPCR. (C) Western blot analysis was performed to detect the expression levels of key EMT markers after HBDSP overexpression. (D) Densitometric quantification of EMT key proteins levels from three independent Western blot experiments, including the representative blot in panel (C). Band intensities were normalized to GAPDH. (E) The expression levels of E‐cadherin (red) and Vimentin (green) were detected by IF following HBDSP overexpression. Nuclei were counterstained with DAPI (blue) (scale bars, 75 μm). (F) Quantification of fluorescence intensity for E‐cadherin and Vimentin in HepG2 and Huh7 cells overexpressing HBDSP from three independent IF assays, including the representative images shown in (E). (G) The relative migration abilities of HepG2 and Huh7 cells overexpressing HBDSP were analyzed using a wound‐healing assay by evaluating their abilities to close a scratch wound created in a monolayer of confluent cells (magnification, ×50). (H) Quantification of the scratch wound closure distances in HepG2 and Huh7 cells overexpressing HBDSP from three independent wound‐healing assays, including the representative images shown in (G). (I) The invasion abilities of HepG2 and Huh7 cells overexpressing HBDSP were assessed using a Matrigel invasion assay (magnification, ×200). (J) Quantification of the number of invasive HepG2 and Huh7 cells overexpressing HBDSP from the three independent Matrigel invasion assays, including the representative images shown in (I). Data are presented as the mean ± SD from three independent experiments. *p* < 0.05 was considered statistically significant compared with the control.

In the present study, an inverted fluorescence microscope was used to examine morphological changes in HepG2 and Huh7 cells. Upon HBDSP overexpression, both HepG2 and Huh7 cells underwent phenotypic changes characteristic of EMT, transitioning from an irregular, flattened shape with few pseudopodia to an elongated, fibroblast‐like morphology (Figure 1A). These results suggest that HBDSP may induce EMT in HepG2 and Huh7 cells. Consistent with these morphological changes, HBDSP‐overexpressed HepG2 and Huh7 cells exhibited decreased expression of the epithelial marker E‐cadherin, along with upregulated expression of the mesenchymal markers N‐cadherin and Vimentin, and the EMT‐associated transcription factor Snail, as confirmed by qPCR, Western blot, and IF assays (Figure 1B–F). Together with the observed morphological changes, these alterations in EMT marker expression support the notion that HBDSP may induce EMT in hepatoma cells.

In parallel with the observed EMT‐related changes, wound‐healing and Matrigel invasion assays were performed to assess cell migration and invasion. As shown in Figure 1G–J, HBDSP enhanced the migratory and invasive abilities of HepG2 and Huh7 cells. Combined with the observed EMT‐like morphological changes and altered expression of EMT markers, these findings suggest that HBDSP may facilitate tumor cell dissemination by promoting EMT and enhancing cell migration and invasion in vitro.

### 3.2 HBDSP may Promote EMT by Upregulating YAP and Its Downstream Genes

**Figure 2:**
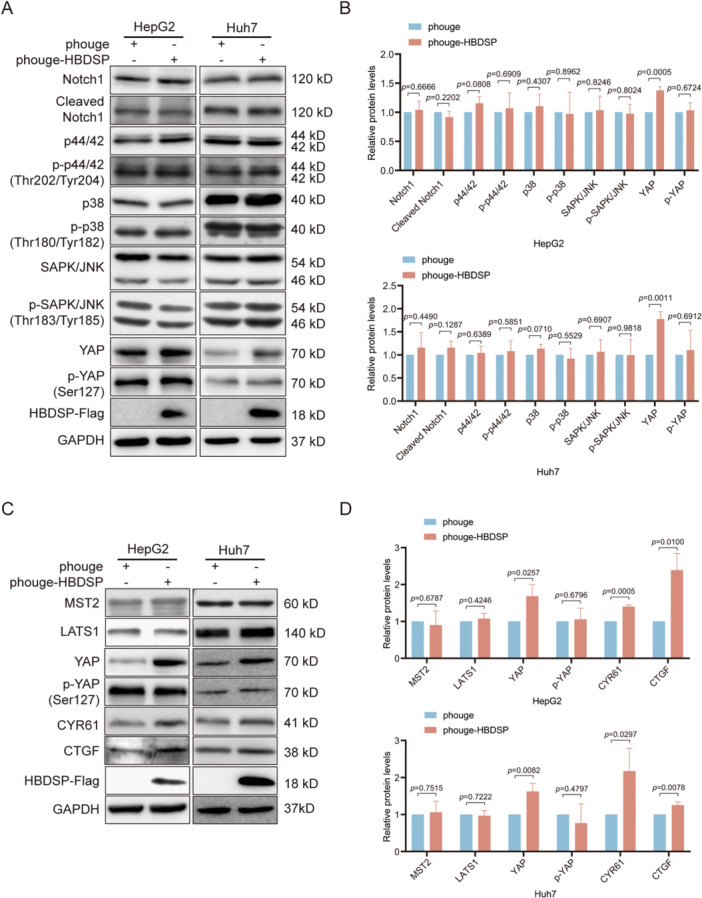
HBDSP overexpression upregulates YAP and its downstream genes in HepG2 and Huh7 cells. (A) Protein expression levels of key EMT‐related signaling components were assessed by Western blot following HBDSP overexpression. (B) Densitometric quantification of protein levels from three independent Western blot experiments, including the representative blot in panel (A). Band intensities were normalized to GAPDH. (C) The expression levels of proteins involved in the Hippo signaling pathway were detected by Western blot following HBDSP overexpression. (D) Densitometric quantification of protein levels from three independent Western blot experiments, including the representative blot in panel (C). Band intensities were normalized to GAPDH. Data are presented as the mean ± SD from three independent experiments. *p* < 0.05 was considered statistically significant compared with the control. *p* > 0.05 was considered not significant.

To identify the signaling pathways potentially involved in HBDSP‐induced EMT and cell motility, we evaluated the expression and phosphorylation levels of representative proteins in several EMT‐related signaling pathways. Western blot analysis revealed that HBDSP overexpression had no significant effects on the total or cleaved levels of Notch1, nor the total or phosphorylated levels of p44/42 (Thr202/Tyr204), p38 (Thr180/Tyr182), or SAPK/JNK (Thr183/Tyr185) (Figure 2A,B). Notably, HBDSP specifically upregulated the total protein levels of YAP without affecting its phosphorylation at Ser172 (Figure 2A,B). Since YAP activity is commonly regulated through phosphorylation‐dependent cytoplasmic retention in the Hippo signaling pathway, the elevated total YAP, together with an unchanged p‐YAP (phosphorylated YAP) levels, suggests a shift that may favor its nuclear localization and transcriptional activity.

To further investigate this possibility, we next examined the expression levels of upstream Hippo regulators (MST2 and LATS1) and downstream YAP target genes (CYR61 and CTGF). We found that HBDSP had no significant effects on MST2 (mammalian sterile 20‐like kinase 2) or LATS1 (large tumor suppressor kinase 1) expression, but increased the expression levels of CYR61 (cysteine‐rich protein 61) and CTGF (connective tissue growth factor) (Figure 2C,D). These results indicate that HBDSP may promote EMT and cell motility by enhancing YAP expression, independent of upstream kinase regulation.

### 3.3 HBDSP Upregulates YAP Expression by Transactivating the −542 to −259 nt Region of the *YAP* Promoter Independent of Protein–Protein Interactions

**Figure 3:**
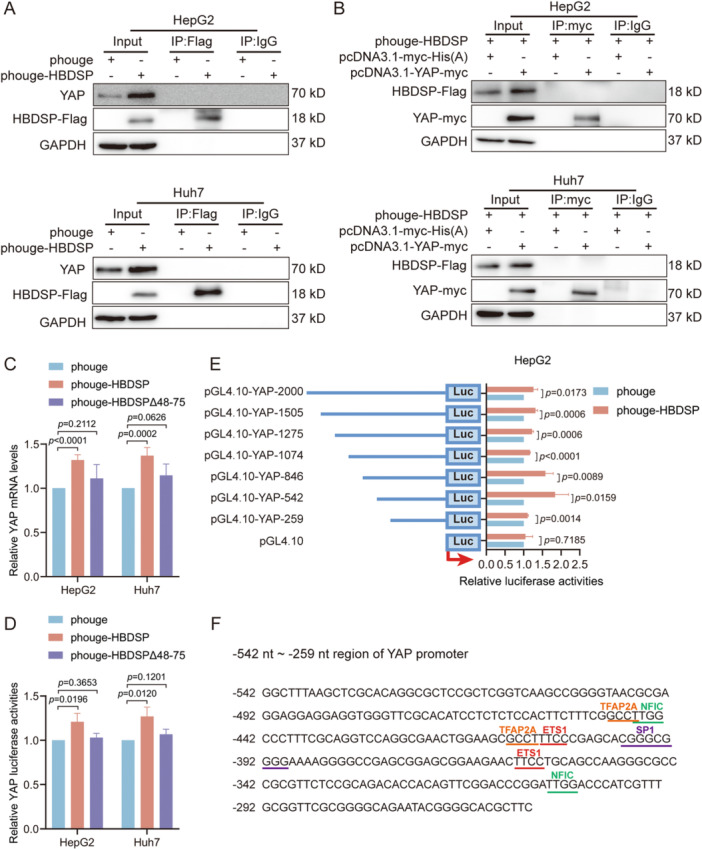
HBDSP transactivates the −542 to −259 nt region of the YAP promoter without interaction. (A) The interaction between HBDSP and YAP was detected by a co‐IP assay after transient transfection with phouge‐HBDSP alone. (B) Co‐IP analysis was performed following co‐transfection with phouge‐HBDSP and pcDNA3.1‐YAP‐myc to further assess the interaction between HBDSP and YAP. (C) The levels of YAP mRNA were quantified by qPCR following transfection with phouge‐HBDSP, phouge‐HBDSPΔ48‐75, or the phouge vector. GAPDH served as an internal control for normalization. (D) The activities of YAP promoter were evaluated using a luciferase reporter assay after co‐transfection with pGL4.10‐YAP‐2000 and either phouge‐HBDSP, phouge‐HBDSPΔ48‐75, or the phouge vector. (E) Luciferase reporter assays were performed to assess the activities of a series of YAP promoter constructs with 5′‐deletion after co‐transfection with phouge‐HBDSP. (F) Putative transcription factors and their binding sites within the −542 to −259 nt region of the YAP promoter were predicted using PROMO, JASPAR, and Gene Regulation databases, and are underlined in the nucleotide sequences. Data are presented as the mean ± SD from three independent experiments. *p* < 0.05 was considered statistically significant compared with the control. *p* > 0.05 was considered not significant.

To further explore the mechanism by which HBDSP upregulates YAP expression, co‐IP assays were performed in HBDSP‐overexpressed HepG2 and Huh7 cells. However, no interaction between HBDSP and YAP was detected under these conditions (Figure 3A). To ensure sufficient protein levels and optimize the detection of potential protein–protein interactions, additional co‐IP assays were conducted in HepG2 and Huh7 cells co‐transfected with pcDNA3.1‐YAP‐myc and phouge‐HBDSP constructs. Consistent with the previous results, no interaction between HBDSP and YAP was observed under these optimized conditions (Figure 3B). These findings suggest that HBDSP may regulate YAP expression through another mechanism that is undetectable by co‐IP.

Subsequently, we assessed *YAP* transcription levels in HepG2 and Huh7 cells overexpressing HBDSP. qPCR analysis revealed that HBDSP increased *YAP* mRNA levels through its transactivating domain (Leu48 to Gln75) (Figure 3C). Moreover, the transcriptional activities of the *YAP* promoter were further evaluated utilizing a luciferase reporter assay. As shown in Figure 3D, HBDSP activated the *YAP* promoter via its transactivating domain, causing an increase in *YAP* gene transcription.

To identify the *YAP* promoter regions involved in HBDSP‐mediated transcriptional regulation, a series of 5′‐deletion constructs were co‐transfected with the phouge‐HBDSP plasmid into HepG2 cells. As shown in Figure 3E, HBDSP overexpression enhanced the relative luciferase activities of all *YAP* promoter constructs compared to the empty vector control. Notably, the pGL4.10‐YAP‐542 construct exhibited the highest luciferase activities, whereas the pGL4.10‐YAP‐259 construct displayed the lowest luciferase activities. These results suggest that the −542 to −259 nt region is critical for the full transcriptional activation of the *YAP* promoter by HBDSP, and may contain key regulatory elements mediating this effect.

Based on these findings, we next sought to identify potential HBDSP‐responsive cis‐acting elements within this critical region of the *YAP* promoter. The −542 to −259 nt region was analyzed using the PROMO, JASPAR, and Gene Regulation databases. Cross‐referencing the prediction results revealed four putative transcription factors (TFAP2A, NFIC, ETS1, and SP1), along with seven corresponding binding sites (Figure 3F).

### 3.4 HBDSP Activates the *YAP* Promoter by Facilitating SP1/ETS1 Nuclear Translocation and Enhancing Their Binding to the −542 to −259 nt Region

**Figure 4:**
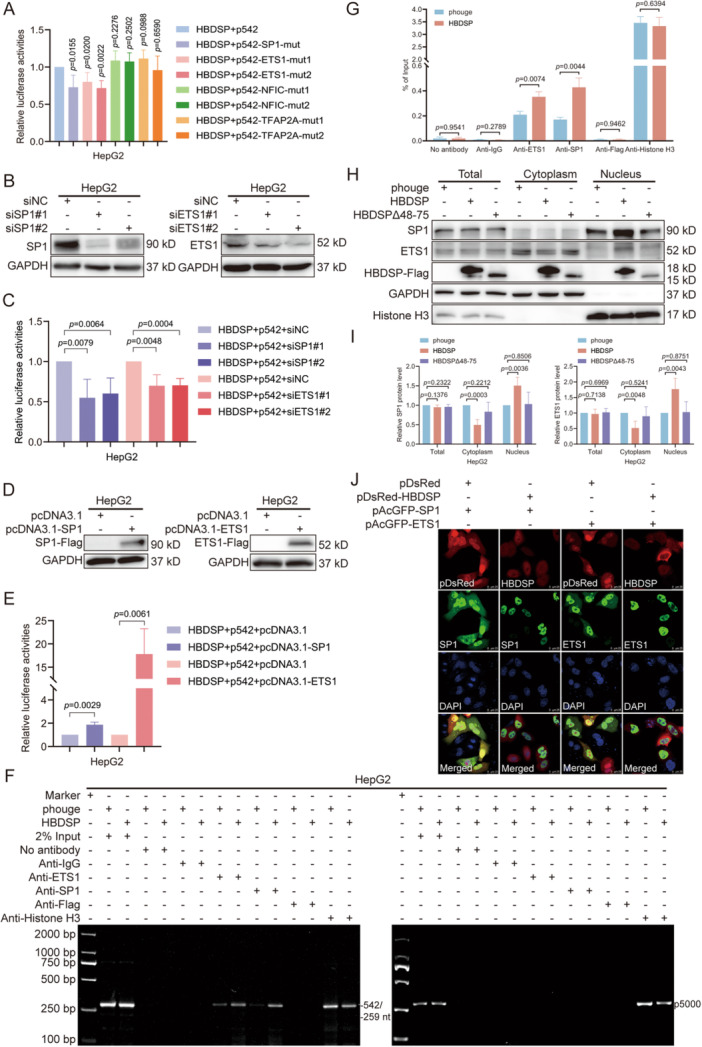
HBDSP enhances SP1 and ETS1 binding to the YAP promoter and promotes their nuclear translocation. (A) The relative luciferase activities of YAP promoter constructs containing mutations in the binding sites for SP1, ETS1, NFIC, or TFAP2A were compared to the wild‐type YAP promoter construct p542 using a luciferase reporter assay after HBDSP overexpression. (B) The knockdown efficiency of SP1 and ETS1 was assessed using the Western blot analysis following siRNA transfection. (C) The relative luciferase activities of the YAP promoter were evaluated using a luciferase reporter assay after co‐transfection of phouge‐HBDSP with siSP1#1, siSP1#2, siETS1#1, or siETS1#2, compared to the siNC control. (D) Overexpression of SP1 and ETS1 was confirmed by the Western blot assay following transfection with respective SP1 and ETS1 expression constructs. (E) Luciferase reporter assays were used to evaluate the relative luciferase activities of the YAP promoter after co‐transfection of phouge‐HBDSP with SP1 or ETS1 expression constructs, compared to the empty vector control. (F) ChIP analysis was performed in HepG2 cells transfected with phouge or phouge‐HBDSP to evaluate the effects of HBDSP on the binding of SP1 and ETS1 to the YAP promoter *in vivo*. The immunoprecipitated DNA was amplified by PCR using primers targeting the −542 to −259 nt region of the YAP promoter and visualized by agarose gel electrophoresis. As a negative control, PCR was also conducted using primers targeting the −5000 to −4717 nt region of the promoter. All amplified fragments were 284 bp in length. IgG served as the negative control for immunoprecipitation, while input DNA and Histone H3 served as the positive controls. (G) The amount of immunoprecipitated DNA was quantified by qPCR. IgG was used as the negative control for immunoprecipitation, and input DNA and Histone H3 were used as the positive controls. Data are expressed as relative enrichment compared to the input control. (H) Total, cytoplasmic, and nuclear proteins were extracted from HepG2 cells transfected with phouge, phouge‐HBDSP, or phouge‐HBDSPΔ48‐75. The expression and subcellular localization of SP1 and ETS1 were examined by Western blot. GAPDH and Histone H3 served as loading controls for the cytoplasmic and nuclear fractions, respectively. (I) Densitometric quantification of SP1 and ETS1 protein levels from three independent Western blot experiments, including the representative blot in panel (H). Band intensities were normalized to GAPDH (for total and cytoplasmic fractions) or Histone H3 (for nuclear fractions). (J) The subcellular distribution of SP1 and ETS1 in HepG2 cells was examined by confocal microscopy following co‐transfection with pDsRed or pDsRed‐HBDSP and pAcGFP‐SP1 or pAcGFP‐ETS1 (scale bars, 25 μm). Data are presented as the mean ± SD from three independent experiments. *p* < 0.05 was considered statistically significant compared with the control. *p* > 0.05 was considered not significant. Abbreviations: HBDSP, phouge‐HBDSP; HBDSPΔ48‐75, phouge‐HBDSPΔ48‐75; p542, pGL4.10‐YAP‐542; p542‐SP1‐mut1, pGL4.10‐YAP‐542‐SP1‐mut1; p542‐ETS1‐mut1, pGL4.10‐YAP‐542‐ETS1‐mut1; p542‐ETS1‐mut2, pGL4.10‐YAP‐542‐ETS1‐mut2; p542‐NFIC‐mut1, pGL4.10‐YAP‐542‐NFIC‐mut1; p542‐NFIC‐mut2, pGL4.10‐YAP‐542‐NFIC‐mut2; p542‐TFAP2A‐mut1, pGL4.10‐YAP‐542‐TFAP2A‐mut1; p54‐TFAP2A‐mut2, pGL4.10‐YAP‐542‐TFAP2A‐mut2.

To verify whether these predicted binding sites are functionally necessary for HBDSP‐mediated activation of the *YAP* promoter, seven mutant constructs were generated. Luciferase reporter assays showed that mutations in the SP1 and ETS1 binding sites reduced promoter activities compared with the wild‐type construct (pGL4.10‐YAP‐542, abbreviated as p542), whereas mutations in the NFIC and TFAP2A binding sites had no significant effects (Figure 4A), indicating that HBDSP promotes *YAP* transcription through SP1 and ETS1. Subsequently, knockdown of *SP1* or *ETS1* using specific siRNAs reduced promoter activities compared with the siNC control in HBDSP‐overexpressed HepG2 cells (Figure 4B,C). Conversely, SP1 or ETS1 overexpression enhanced HBDSP‐induced promoter activation compared with the empty vector control (Figure 4D,E).

The binding of SP1 and ETS1 to the *YAP* promoter was examined in HepG2 cells by ChIP (in vivo) and EMSA (in vitro). As shown in Figure 4F (ChIP‐PCR) and 4 G (ChIP‐qPCR), enrichment of the −542 to −259 nt region of the *YAP* promoter was detected in the SP1 and ETS1 immunoprecipitates, but not in the IgG or no‐antibody controls. Notably, HBDSP overexpression significantly enhanced SP1‐ and ETS1‐mediated enrichment of the *YAP* promoter region relative to the control. Consistent with the ChIP data, EMSA (Figure S1) analyses demonstrated that overexpression of HBDSP enhanced the formation of DNA‐protein complexes corresponding to SP1 and ETS1 binding (lane 7 vs. lane 2). These shifted bands disappeared after adding a 100‐fold excess of unlabeled competitor probes and were only partially diminished by mutated competitor probes, confirming binding specificity (lanes 3, 4, 8, and 9). Furthermore, super‐shifted bands were observed following the addition of SP1‐ or ETS1‐specific antibodies (lanes 5 and 10), further verifying the identities of the bound transcription factors.

The subcellular localization of SP1 and ETS1 was subsequently investigated in HBDSP‐overexpressed HepG2 cells. We observed that HBDSP had no significant effects on the total protein levels of SP1 and ETS1, but reduced their cytoplasmic abundance and increased their nuclear accumulation through its transactivating domain (Figure 4H,I). Consistently, confocal microscopy further confirmed that SP1 and ETS1 signals were predominantly localized in the nucleus in the presence of HBDSP, whereas they were distributed throughout both the cytoplasm and nucleus in the control group (Figure 4J).

### 3.5 HBDSP Induces EMT, Migration, and Invasion in a YAP‐Dependent Manner

**Figure 5:**
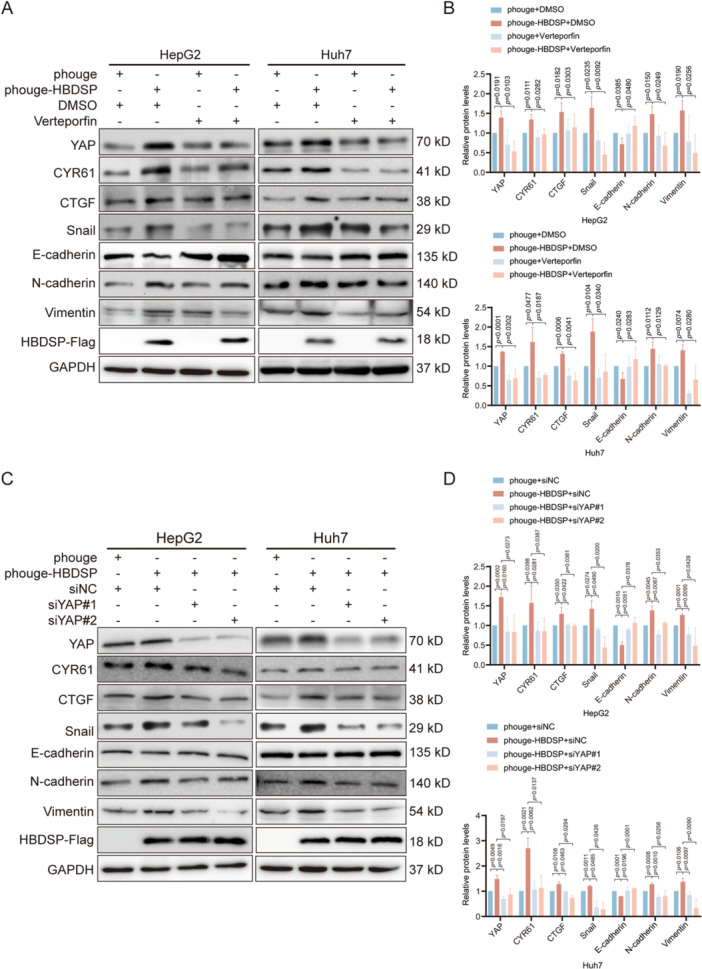
HBDSP induces EMT through a YAP‐dependent mechanism in HepG2 and Huh7 cells. (A) The expression levels of key EMT markers and YAP downstream effectors were detected by Western blot in HepG2 and Huh7 cells following HBDSP overexpression and treatment with Verteporfin, a YAP inhibitor. (B) Densitometric quantification of protein levels from three independent Western blot experiments, including the representative blot in panel (A). Band intensities were normalized to GAPDH. (C) The expression levels of key EMT markers and YAP downstream targets were assessed by Western blot in HepG2 and Huh7 cells after co‐transfection with phouge‐HBDSP or phouge and siRNAs targeting YAP (siYAP#1, siYAP#2) or a negative control siRNA (siNC). (D) Densitometric quantification of proteins levels from three independent Western blot experiments, including the representative blot in panel (C). Band intensities were normalized to GAPDH. Data are presented as the mean ± SD from three independent experiments. *p* < 0.05 was considered statistically significant compared with the control.

We have demonstrated that HBDSP promotes the nuclear translocation of SP1 and ETS1, thereby enhancing their binding to the *YAP* promoter and facilitating *YAP* transcription (Figures 4 and S1). Furtherly, the YAP‐specific inhibitor Verteporfin and *YAP*‐targeting siRNAs were used to inhibit YAP expression in HepG2 and Huh7 cells. Western blot analysis revealed that treatment with 1.5 μM Verteporfin led to a reduction in YAP, CYR61, CTGF, Snail, N‐cadherin, and Vimentin levels, and an increase in E‐cadherin expression compared to the DMSO‐treated control (Figure 5A,B, lane 4 vs. lane 2). Similarly, *YAP* knockdown using siRNAs (siYAP#1 and siYAP#2) downregulated YAP, CYR61, CTGF, Snail, N‐cadherin, and Vimentin, while upregulating E‐cadherin compared to the siNC control (Figure 5C,D, lane 3 vs. lane 2 and lane 4 vs. lane 2).

**Figure 6:**
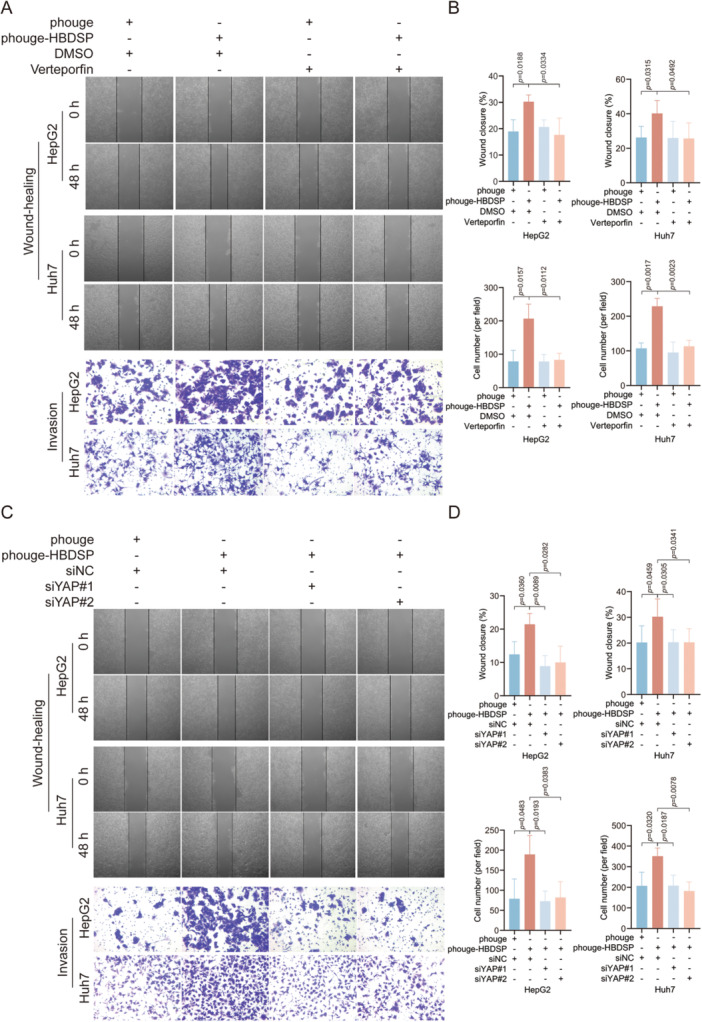
HBDSP promotes the migration and invasion of HepG2 and Huh7 cells in a YAP‐dependent manner. (A) The migratory and invasive abilities of HepG2 and Huh7 cells transfected with phouge‐HBDSP or phouge and treated with Verteporfin or DMSO were analyzed using wound‐healing and Matrigel invasion assays, respectively. Cell motility was evaluated based on the closure of a scratch wound created in a monolayer of confluent cells (magnification, ×50). The invasiveness was assessed by quantifying the number of cells that invaded through the Matrigel (magnification, ×200). (B) Quantification of scratch wound closure distances and invasive cell numbers based on three independent wound‐healing and Matrigel invasion assays. Representative images are shown in (A). (C) The migratory and invasive abilities of HepG2 and Huh7 cells co‐transfected with phouge‐HBDSP or phouge and siYAP#1, siYAP#2, or siNC were similarly analyzed using wound‐healing and Matrigel invasion assays. Cell motility and invasiveness were assessed using the same criteria as described in (A). (D) Quantification of scratch wound closure distances and invasive cell numbers based on three independent wound‐healing and Matrigel invasion assays. Representative images are shown in (C). Data are presented as the mean ± SD from three independent experiments. *p* < 0.05 was considered statistically significant compared with the control.

Wound‐healing and Matrigel invasion assays were also performed following YAP inhibition. The results demonstrated that HBDSP overexpression enhanced cell migration and invasion, while treatment with Verteporfin or transfection with *YAP*‐targeting siRNAs significantly reduced cell migration and invasion (Figure 6A–D). These findings suggest that HBDSP‐induced EMT, migration, and invasion are YAP‐dependent.

### 3.6 HBDSP Promotes YAP‐Dependent EMT, Migration, and Invasion Under HBV‐Positive Conditions

**Figure 7:**
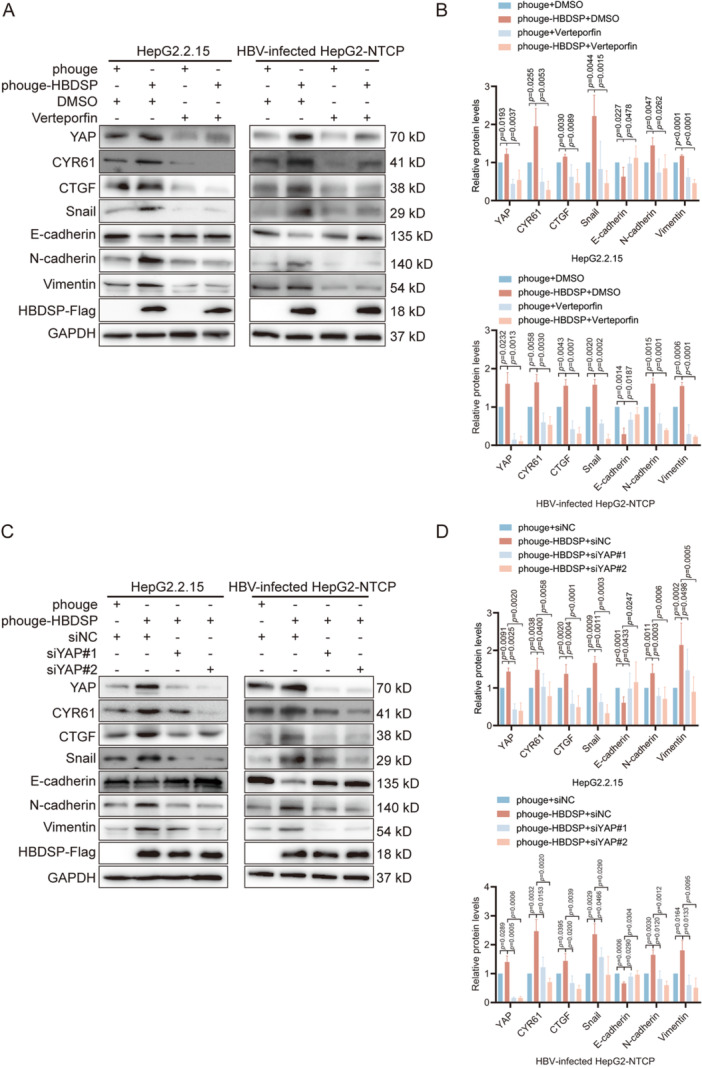
YAP is essential for HBDSP‐mediated EMT in HepG2.2.15 and HBV‐infected HepG2‐NTCP cells. (A) Western blot analysis was performed to examine the expression levels of key EMT markers and YAP downstream effectors in HepG2.2.15 and HBV‐infected HepG2‐NTCP cells following HBDSP overexpression and treatment with Verteporfin, a YAP inhibitor. (B) Densitometric quantification of protein levels from three independent Western blot experiments, including the representative blot in panel (A). Band intensities were normalized to GAPDH. (C) Western blot analysis was used to detect the expression levels of key EMT markers and YAP downstream targets in HepG2.2.15 and HBV‐infected HepG2‐NTCP cells co‐transfected with either phouge‐HBDSP or phouge and siRNAs targeting YAP (siYAP#1, siYAP#2), or a negative control siRNA (siNC). (D) Densitometric quantification of protein levels from three independent Western blot experiments, including the representative blot in panel (C). Band intensities were normalized to GAPDH. Data are presented as the mean ± SD from three independent experiments. *p* < 0.05 was considered statistically significant compared with the control.

**Figure 8:**
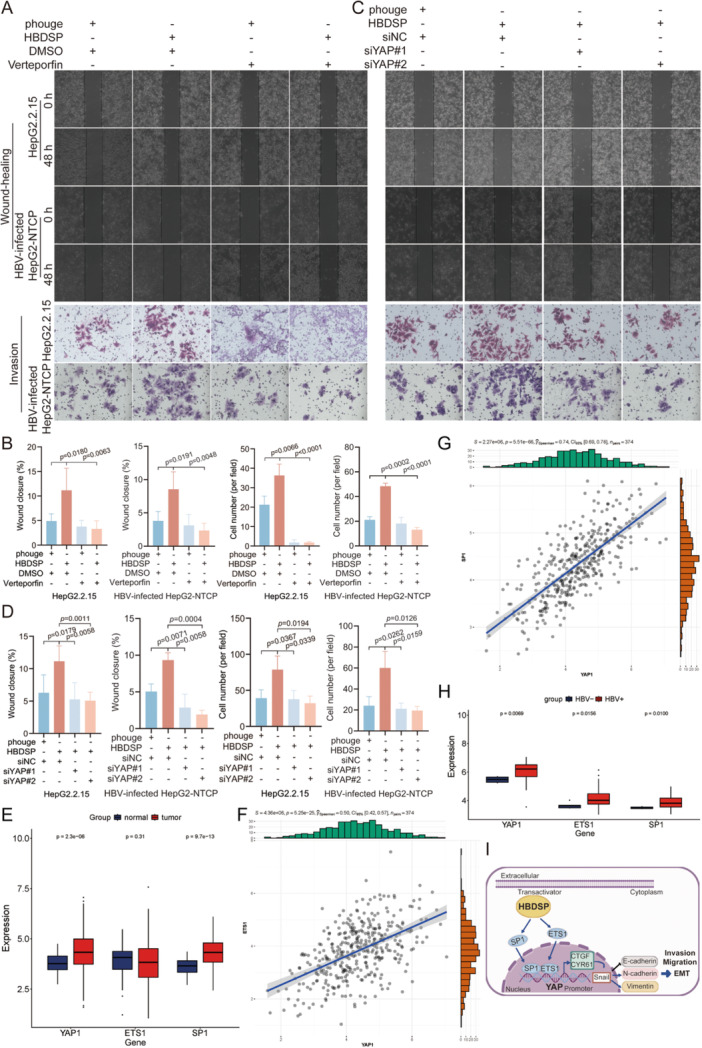
YAP‐dependent migration and invasion in HBV‐positive hepatoma cells and clinical correlation of the SP1/ETS1 and YAP. (A) Wound‐healing and Matrigel invasion assays were used to evaluate the migratory and invasive capacities of HepG2.2.15 and HBV‐infected HepG2‐NTCP cells transfected with phouge‐HBDSP (abbreviated as HBDSP) or phouge and treated with Verteporfin or DMSO, respectively. Cell motility was assessed based on the closure of scratch wounds in confluent cell monolayers (magnification, ×50), while invasiveness was determined by quantifying the number of invading cells (magnification, ×200). (B) Quantification of scratch wound closure distances and invasive cell numbers based on three independent wound‐healing and Matrigel invasion assays. Representative images are shown in (A). (C) Wound‐healing and Matrigel invasion assays were performed to analyze the migratory and invasive abilities of HepG2.2.15 and HBV‐infected HepG2‐NTCP cells co‐transfected with either phouge‐HBDSP or phouge and siYAP#1, siYAP#2, or siNC. Cell motility and invasiveness were assessed as described in (A). (D) Quantification of scratch wound closure distances and invasive cell numbers based on three independent wound‐healing and Matrigel invasion assays. Representative images are shown in (C). Data are presented as the mean ± SD from three independent experiments. *p* < 0.05 was considered statistically significant compared with the control. (E) RNA‐seq data from the TCGA‐LIHC cohort were analyzed to compare the expression levels of YAP1, SP1, and ETS1 between HCC tissues (tumor) and matched adjacent non‐tumor liver samples (normal). (F) Spearman correlation analysis of YAP1 and ETS1 expression in HCC tissues from the TCGA‐LIHC cohort. (G) Spearman correlation analysis of YAP1 and SP1 expression in HCC tissues from the TCGA‐LIHC cohort. (H) Expression levels of YAP1, SP1, and ETS1 in liver tissues from patients with CHB (HBV‐positive) and normal controls (HBV‐negative) in the GEO data set GSE83148. Statistical significance and Spearman's correlation coefficients are indicated in the respective panels. (I) Schematic diagram of HBDSP promoting YAP‐driven EMT, migration, and invasion in hepatoma cells. In hepatoma cells, HBDSP promotes the nuclear translocation of SP1 and ETS1, enhancing their binding to the YAP promoter and activating YAP transcription. YAP activation upregulates CTGF, CYR61, and Snail, which suppresses E‐cadherin and increases N‐cadherin and Vimentin expression, thereby inducing EMT and enhancing cell migration and invasion.

To assess the regulatory role of HBDSP in EMT, migration, and invasion in HBV‐replicating and HBV‐infected cells, we further investigated its effects in HepG2.2.15 cells, a stable HBV‐expressing hepatoma cell line, and in HBV‐infected HepG2‐NTCP cells. Consistent with the results observed in HepG2 and Huh7 cells, HBDSP overexpression in HepG2.2.15 and HBV‐infected HepG2‐NTCP cells resulted in decreased E‐cadherin levels and increased levels of YAP, CYR61, CTGF, Snail, N‐cadherin, and Vimentin (Figure 7A–D, lane 2 vs. lane 1). Notably, inhibition of YAP by using Verteporfin or *YAP* siRNA reversed these effects (Figure 7A,B, lane 4 vs. lane 2; Figure 7C,D, lane 3 vs. lane 2 and lane 4 vs. lane 2), indicating that YAP is also involved in HBDSP‐induced EMT in HBV‐replicating and HBV‐infected cells. Functional assays further demonstrated that HBDSP enhanced the migratory and invasive abilities of HepG2.2.15 and HBV‐infected HepG2‐NTCP cells, while these effects were significantly attenuated upon YAP inhibition (Figure 8A–D). Collectively, these findings reveal that HBDSP facilitates EMT, migration, and invasion through YAP signaling in both HBV‐negative and HBV‐positive hepatoma cells, which indicates that YAP plays a critical role in HBV‐associated malignant phenotypes.

### 3.7 The SP1/ETS1 and YAP Expression Are Positively Correlated in HCC and Upregulated in HBV‐Associated Liver Tissues

To further validate the clinical relevance of these findings, we analyzed publicly available transcriptomic datasets. In the TCGA‐LIHC cohort, *YAP1* and *SP1* mRNA levels were significantly upregulated in HCC tissues compared with adjacent non‐tumor liver samples (Figure 8E). Although *ETS1* expression displayed considerable heterogeneity, correlation analysis revealed positive correlations between *ETS1* and *YAP1*, as well as between *SP1* and *YAP1* (Figure 8F,G), suggesting that these factors may be coordinately regulated at the transcript level in HCC. We further analyzed the GEO data set GSE83148 to explore the relationship with HBV infection. Notably, *YAP1*, *ETS1*, and *SP1* expression levels were significantly elevated in liver biopsy samples from CHB patients compared with controls (Figure 8H), indicating that the expression pattern of *SP1/ETS1* and *YAP* may be modulated by HBV infection status. Collectively, these findings provide supportive clinical correlative evidence that the coordinated expression of *SP1/ETS1* and *YAP* may also be relevant in HBV‐associated liver disease and hepatocarcinogenesis.

## 4 Discussion

Although wtHBV has long been regarded as the primary agent driving the progression and pathogenesis of liver diseases, growing evidence indicates that spHBV, generated during chronic infection, also contributes to these processes [7]. Clinical studies have demonstrated a progressive increase in spHBV levels during liver disease progression, with a positive association to the severity of hepatic injury. For instance, studies by Soussan et al. and Redelsperger et al. demonstrated that the levels of spHBV were significantly higher in patients with more advanced liver disease or severe fibrosis than those with milder disease [24, 25]. Moreover, spHBV levels have been shown to annually increase prior to the clinical diagnosis of HCC, and are significantly elevated in tumor tissues relative to adjacent non‐tumorous tissues [6, 26]. In addition, HCC patients with HBV infection exhibit a higher risk of metastasis and recurrence compared to those without HBV infection [27, 28]. Considering that HBV alternative splicing is a common event during chronic HBV infection, these findings underscore the potential involvement of spliced variants in HCC initiation and progression. Nevertheless, the biological functions of these spliced variants and their encoded proteins remain incompletely understood.

Among the identified spliced variants, sp1 is the most frequently detected [29]. Its encoded protein, HBSP, has been shown to promote liver disease through multiple mechanisms, including cytotoxic T lymphocyte (CTL)‐mediated liver damage, severity of fibrosis, and Bcl‐2/Bcl‐xl‐induced apoptosis [30, 31, 32]. In addition, HBSP interferes with coagulation, enhances oncogenic susceptibility, and facilitates the proliferation and invasive metastasis of hepatoma cells by interactions with various host proteins, such as fibrinogen gamma chain, microsomal epoxide hydrolase, and cathepsin B [10, 33, 34]. Notably, Maslac et al. reported that sp7, sp8, and sp13 are also commonly detected in the serum of patients across distinct stages of chronic HBV infection [35]. Among them, sp7 has been demonstrated to promote wtHBV replication [14], and its encoded protein, HBDSP, is able to affect the expression of host oncogenes and virulence genes through transactivation, thereby potentially contributing to HBV pathogenesis [13]. Importantly, our previous studies have confirmed the objective existence of HBDSP protein by detecting it with a specific anti‐HBDSP antibody in both transiently transfected Huh7 cells and HBV‐replicating cell models, including HepG2.2.15, HepAD38, and HepG2‐NTCP [13, 15]. However, the functional roles of HBDSP remain largely unexplored. The present study aimed to further clarify the biological functions and underlying mechanisms of HBDSP in hepatoma cells, and to explore how these effects might relate to the progression of HBV‐associated HCC.

In this study, we provide the first evidence that HBDSP promotes the EMT, migration and invasion of hepatoma cells. Distant metastasis is a hallmark of malignancy, and accounts for over 90% of cancer‐related deaths [36]. Patients with HBV‐associated HCC generally exhibit a poorer prognosis due to high rates of recurrence and metastasis [37]. EMT is a critical mechanism in metastatic progression of tumors, enabling tumor cells to escape from primary sites and disseminate to regional or distant organs [38]. Previous investigations have revealed that EMT is implicated in HBV‐induced HCC metastasis, and HBV infection enhances the invasive capacity of HCC cells by promoting EMT [39, 40, 41]. Therefore, clarifying the molecular mechanisms underlying metastasis and invasion in HBV‐associated HCC is vital for developing novel therapeutic strategies. Our findings demonstrate that overexpression of HBDSP triggered EMT phenotype transition by transforming HepG2 and Huh7 cells from a typical, flattened epithelial morphology into an elongated, irregular fibroblast‐like shape, accompanied by increased expression of mesenchymal markers (N‐cadherin and Vimentin) and the EMT‐related transcription factor Snail, along with decreased expression of the epithelial marker E‐cadherin. Moreover, overexpression of HBDSP enhanced the migratory and invasive abilities of hepatoma cells. Consistently, in the HBV‐replicating HepG2.2.15 cell line and HBV‐infected HepG2‐NTCP cell line, HBDSP also promoted EMT, migratory, and invasive capacities, suggesting that this effect is preserved in the context of viral replication and infection. This finding highlights that HBDSP may link HBV with the enhanced metastatic potential of hepatoma cells. These changes suggest a potential role of HBDSP in regulating tumor metastasis, a biological function that has not been deeply explored before.

Mechanistically, HBDSP specifically upregulated the expression of YAP and its downstream targets CYR61 and CTGF, without altering the phosphorylation status of YAP or the expression of upstream regulators (MST2 and LATS1) in the typical Hippo pathway. YAP plays a central role in the EMT and metastatic progression of various cancers, including HCC. In human HCC cell lines and tissues, YAP is highly expressed and significantly correlates with the levels of EMT characteristic markers [42, 43]. Overexpression of YAP has been shown to promote the migration and invasion of hepatoma cells [44], whereas YAP knockdown suppresses these behaviors by inhibiting EMT [42]. Furthermore, YAP drives EMT through transcriptional upregulation of Snail, consequently accelerating cell migration and invasion [45]. These findings emphasize the pivotal role of YAP in regulating EMT and tumor metastasis. Based on this evidence, we hypothesized that YAP may function as a key effector in HBDSP‐mediated EMT, migration, and invasion.

To verify the proposed mechanism, YAP‐specific siRNA and pharmacological inhibitor Verteporfin were employed in this study to disrupt YAP function. Both interventions significantly reversed the HBDSP‐induced EMT phenotype and the enhanced migratory and invasive capabilities of HepG2, Huh7, HepG2.2.15, and HBV‐infected HepG2‐NTCP cells, thereby confirming the critical role of YAP in this process. These findings identify YAP as a central mediator of HBDSP‐driven malignant phenotypes in hepatoma cells in both HBV‐positive and negative contexts. Furthermore, previous studies have shown that the combination of Verteporfin and sorafenib enhances antitumor efficacy [43]. These findings underscore the critical role of the HBDSP‐YAP axis in promoting malignancy and warrant further investigation into its therapeutic relevance.

Furthermore, considering that HBDSP functions as a pleiotropic viral transactivator, we further investigated the molecular mechanism by which HBDSP upregulates YAP expression. Our results indicate that HBDSP promotes *YAP* transcription through its transactivation domain located at amino acid residues 48–75, rather than via interaction with the YAP protein. Mechanistically, this effect is mediated by enhancing the nuclear translocation of the transcription factors ETS1 and SP1, thereby facilitating their binding to the *YAP* promoter region spanning −542 to −259 nt. This binding activity leads to increased transcriptional activation of *YAP*. Notably, to our knowledge, the present study is the first to reveal that ETS1 specifically binds to the *YAP* promoter region and to confirm its functional relevance in *YAP* transcriptional activation, providing novel insights into its transcriptional regulation. Moreover, we revealed that the SP1 binding region within the *YAP* promoter differs from that reported in previous studies [46], suggesting that SP1 may bind to multiple regions of the *YAP* promoter to regulate transcription. Although this study establishes a functional link between HBDSP expression and the enhanced nuclear localization of ETS1 and SP1, the precise upstream mechanism governing this process remains to be elucidated, representing an important direction for future investigation. Collectively, these findings suggest that HBDSP regulates *YAP* transcription through unique cis‐regulatory elements, providing novel insights into how viral proteins modulate host oncogene expression.

Notably, the mechanisms by which HBDSP exerts its effects appear to differ from those of HBSP. While HBSP contributes to HCC development and progression primarily through protein‐protein interactions with host proteins, HBDSP predominantly influences malignant‐related phenotypes through transcriptional activation of host genes. Whether HBDSP also engages in direct protein‐protein interactions remains an open question for future investigation. This study primarily relies on an HBDSP overexpression system to reveal its novel role in activating the YAP pathway. However, we recognize that future studies comparing wtHBV genomes with those incapable of producing HBDSP will be crucial for clarifying its specific role in HBV‐related pathogenic mechanisms in a more physiologically relevant context. Furthermore, our previous study demonstrated that HBDSP promotes apoptosis and enhances HBV progeny production through *p53* activation mediated by ETS1, GATA2, and YY1 [15]. That work primarily focused on the pro‐apoptotic signaling triggered by HBDSP in hepatoma cells expressing wild‐type p53, uncovering a pathway that links viral proteins to cell death and viral release. The present study uncovers another novel cancer‐promoting mechanism, in which HBDSP drives EMT, migration, and invasion of hepatoma cells via transactivation of *YAP* mediated by ETS1 and SP1. Interestingly, ETS1 emerges as a shared transcription factor in both studies. As a highly versatile transcription factor, its functional output is strongly dependent on interacting partners and the cellular context [47]. These observations suggest that HBDSP may modulate transcriptional complexes involving ETS1 to regulate distinct downstream gene expression, thereby exerting diverse biological functions. Together, these findings emphasize the pleiotropic nature of HBDSP, capable of manipulating diverse host transcriptional networks to orchestrate distinct biological outcomes.

Although the cancer‐promoting effects of HBDSP observed in our experiments were statistically significant, we acknowledge that the magnitude of these effects always appeared relatively modest. This is consistent with our previous studies, in which HBDSP exhibited moderate transactivation activity on host and viral gene promoters [13] and promoted p53‐dependent apoptosis via nuclear translocation of transcription factors ETS1, GATA2, and YY1 [15]. Despite the relatively small fold changes observed, these effects were reproducible and biologically meaningful, underscoring the functional relevance of HBDSP in hepatoma cells. This modest effect may reflect the progressive and subtle nature of HBDSP‐induced HBV‐associated hepatocarcinogenesis, which typically requires long‐term accumulation and evolution in vivo. Notably, similarly modest effects have also been reported for other HBV‐encoded proteins, such as HBx [48] and HBSP [49]. HBx‐mediated upregulation of proliferation‐ and migration‐related genes rarely exceeded twofold, and HBSP‐induced modulation of the anti‐apoptotic gene FLIP_L_ was less than 1.5‐fold. Collectively, these observations suggest that HBV‐derived proteins may contribute to hepatocarcinogenesis not through strong and acute transformation but via subtle and persistent modulation of host cellular processes. Therefore, it is important to consider the chronic and multifactorial nature of HBV‐host interactions when interpreting the functional significance of such modest yet reproducible effects.

In summary, this study uncovers a novel mechanism by which the HBV spliced variant sp7‐encoded protein HBDSP potentiates the malignant phenotypes of hepatoma cells by transcriptionally activating YAP. Specifically, HBDSP facilitates the nuclear translocation of transcription factors SP1 and ETS1, enhancing their binding to the *YAP* promoter, which activates *YAP* transcription and upregulates its expression. YAP activation further upregulates its downstream target genes CTGF and CYR61 and elevates the levels of the EMT transcription factor Snail. The increased expression of Snail suppresses the epithelial marker E‐cadherin and promotes the upregulation of mesenchymal markers N‐cadherin and Vimentin, further driving EMT and enhancing cell migration and invasion (Figure 8I). Together with bioinformatic evidence from independent clinical cohorts demonstrating elevated *YAP1*, *SP1*, and *ETS1* expression in HBV‐associated liver disease, these findings further support the potential clinical relevance of the HBDSP‐SP1/ETS1‐YAP regulatory axis. This finding not only expands our understanding of the pathogenic role of HBV spliced variants, but also highlights HBDSP‐mediated activation of YAP as a promising candidate for future investigation into targeted therapies.

## Author Contributions

**Xiazhen Xu:** conceptualization, investigation, methodology, visualization, writing – original draft. **Yibin Peng:** data curation, formal analysis, and investigation. **Yuxin Ping:** investigation, validation, and visualization. **Tiantian Shao:** formal analysis and validation. **Qiong Wu:** methodology and software. **Lu Zhang:** data curation and software. **Xu Lin:** supervision, writing – review and editing. **Changxi Yu:** supervision, writing – review and editing. **Wannan Chen:** conceptualization, funding acquisition, resources, methodology, supervision, project administration, writing – review and editing.

## Conflicts of Interest

The authors declare no conflicts of interest.

## Supporting information


**Figure S1**: HBDSP enhances SP1 and ETS1 binding to the YAP promoter in vitro.

**Table S1:** The sequences of wild‐type and mutated oligonucleotide corresponding to the predicted SP1, ETS1, NFIC, and TFAP2A binding sites.
**Table S2:** Sequences of primers used in plasmid construction.
**Table S3:** Sequences of the siRNA oligo used in RNA interference.
**Table S4:** Sequences of primers used in qPCR.
**Table S5:** Sequences of the probes used in EMSA.

## Data Availability

The authors confirm that all data supporting the findings of this study are available within the article and its Supplementary Materials. The data are available from the corresponding author upon reasonable request.
