# Supplementary figures and images for: Hepatitis B Doubly Spliced Protein (HBDSP) Promotes Epithelial‐Mesenchymal Transition, Migration, and Invasion via SP1/ETS1‐Dependent YAP Activation in Hepatoma Cells
Source: J Med Virol. 2026 Jul 9;98(7):e71046. doi: 10.1002/jmv.71046 (PMC13348490; doi:10.1002/jmv.71046)

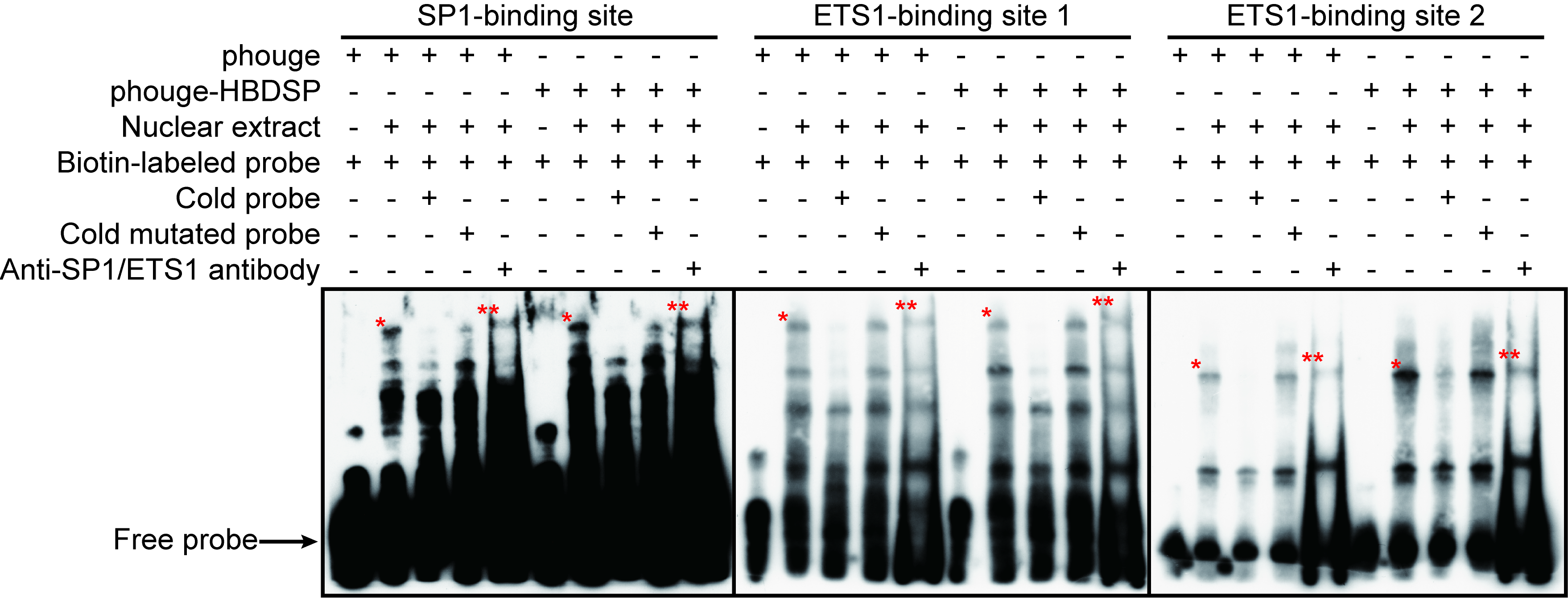

Supplement: Supplementary file 1 — Figure S1: HBDSP enhances SP1 and ETS1 binding to the YAP promoter in vitro. [file JMV-98-e71046-s001.tif]
