# Supplementary material for: Hepatitis B Doubly Spliced Protein (HBDSP) Promotes Epithelial‐Mesenchymal Transition, Migration, and Invasion via SP1/ETS1‐Dependent YAP Activation in Hepatoma Cells
Source: J Med Virol. 2026 Jul 9;98(7):e71046. doi: 10.1002/jmv.71046 (PMC13348490; doi:10.1002/jmv.71046)
Supplement: Supplementary file 2 — Table S1: The sequences of wild‐type and mutated oligonucleotide corresponding to the predicted SP1, ETS1, NFIC, and TFAP2A binding sites. Table S2: Sequences of primers used in plasmid construction. Table S3: Sequences of the siRNA oligo used in RNA interference. Table S4: Sequences of primers used in qPCR. Table S5: Sequences of the probes used in EMSA. [file JMV-98-e71046-s002.pdf]

Supplementary Figure 1. HBDSP enhances SP1 and ETS1 binding to the YAP promoter *in vitro*. EMSA was performed using nuclear extracts from HepG2 cells transfected with phouge or phouge-HBDSP to assess the effects of HBDSP on the binding of SP1 and ETS1 to the YAP promoter *in vitro*. The specific protein-DNA complexes (shifted bands) are marked with single red asterisk, and the super-shifted bands are denoted with double red asterisks.

Supplementary Table 1. The sequences of wild-type and mutated oligonucleotide corresponding to the predicted SP1, ETS1, NFIC, and TFAP2A binding sites.

Plasmids	Recognition sites	Wild sequences (5´-3´)	Mutated sequences (5´-3´)
p542-SP1-mut	-398 ~ -390	CGGGCGGGG	CGttttGGG
p542-ETS1-mut1	-362 ~ -359	TTCC	TTaa
p542-ETS1-mut2	-408 ~ -405	TTCC	TTaa
p542-NFIC-mut1	-446 ~ -443	TTGG	Tcat
p542-NFIC-mut2	-207 ~ -204	TTGG	Tcat
p542-TFAP2A-mut1	-449 ~ -446	GCCT	taaT
p542-TFAP2A-mut2	-412 ~ -409	GCCT	taaT

The bases in lower case represent mutated SP1, EST1, NFIC and TFAP2A recognition sites.

Supplementary Table 2. Sequences of primers used in plasmid construction.

Plasmids	Primers (5´-3´)
pcDNA3.1-	F: CTAGCTAGCGCCACCATGGATCCCGGGCAGCAGCCG
YAP-myc	R: GGGGTACCTAACCATGTAAGAAAGCTTTC
pGL4.10-	F: GGCCTAACTGGCCGGTACCTTACTCTGATCCTTTT
YAP-1505	R: CGAGGCCAGATCTTGATATCCTCGAGCCACTGGTCTGGCGGCTGCG
pGL4.10-	F: GGCCTAACTGGCCGGTACCTAGCATTGTAGACAA
YAP-1275	R: CGAGGCCAGATCTTGATATCCTCGAGCCACTGGTCTGGCGGCTGCG
pGL4.10-	F: GGCCTAACTGGCCGGTACCAAAAAACCCAGAGTG
YAP-1074	R: CGAGGCCAGATCTTGATATCCTCGAGCCACTGGTCTGGCGGCTGCG
pGL4.10-	F: GGCCTAACTGGCCGGTACCAGACGCACTAATTTTTGGGTTG
YAP-846	R: CGAGGCCAGATCTTGATATCCTCGAGCCACTGGTCTGGCGGCTGCG
pGL4.10-	F: GGCCTAACTGGCCGGTACCGGCTTTAAGCTCGCA
YAP-542	R: CGAGGCCAGATCTTGATATCCTCGAGCCACTGGTCTGGCGGCTGCG
pGL4.10-	F: GGCCTAACTGGCCGGTACCGGGCCCCTGCCCGGCT
YAP-259	R: CGAGGCCAGATCTTGATATCCTCGAGCCACTGGTCTGGCGGCTGCG
pcDNA3.1-	F: CTAGCTAGCGCCACCATGAGCGACCAAGATCACTC
SP1	R: GGGGTACCCTACTTGTCGTCATCGTCTTTGTAGTCGAAGCCATT
pAcGFP-SP1	F: CTAGCTAGCGCCACCATGAGCGACCAAGATCACTCC
	R: GGGGTACCGTCTTGTCGTCATCGTCTTTGTAGTCGAAGC

Underlined sequences represent the restriction enzyme recognition sites of vectors.

Supplementary Table 3. Sequences of the siRNA oligo used in RNA interference.

siRNA Name	Gene Accession Number	Sequences (5´-3´)
siYAP#1	NM_001130145.3	sense: GGUGAUACUAUCAACCAAATT
		anti-sense: UUUGGUUGAUAGUAUCACCTT
siYAP#2	NM_001130145.3	sense: CUGCCACCAAGCUAGAUAATT
		anti-sense: UUAUCUAGCUUGGUGGCAGTT
siSP1#1	NM_138473.3	sense: UUGAGUCACCCAAUGAGAATT
		anti-sense: UUCUCAUUGGGUGACUCAATT
siSP1#2	NM_138473.3	sense: GGAUGGUUCUGGUCAAAUACA
		anti-sense: UAUUUGACCAGAACCAUCCUG
siETS1#1	NM_001143820.2	sense: GCAGUUUCUUCUGGAAUUATT
		anti-sense: UAAUUCCAGAAGAAACUGCTT
siETS1#2	NM_001143820.2	sense: GCACCUUCAAGGACUAUGUTT
		anti-sense: ACAUAGUCCUUGAAGGUGCTT
siNC		sense: UUCUCCGAACGUGUCACGUTT
		anti-sense: ACGUGACACGUUCGGAGAATT

Supplementary Table 4. Sequences of primers used in qPCR.

Gene Symbols	Gene Accession Number	Primers (5´-3´)
YAP	NM_001130145.3	F: GAACTCGGCTTCAGGTCCTC
		R: GGTTCATGGCAAAACGAGGG
E-cadherin	NM_004360.5	F: AGCCCCGCCTTATGATTCTCTG
		R: TGCCCCATTCGTTCAAGTAGTCAT
N-cadherin	NM_001792.5	F: GATGAAACGCCGGGATAAAGAAC
		R: GCTGCAGCTGGCTCAAGTCATAG
Vimentin	NM_003380.5	F: TTGAACGCAAAGTGGAATC
		R: AGGTCAGGCTTGGAAACA
Snail	NM_005985.4	F: GACCCCAATCGGAAGCCTAACTAC
		R: AGCCTTTCCCACTGTCCTCATC
GAPDH	NM_002046.7	F: CTCATGACCACAGTCCATGC
		R: CAGTGAGCTTCCCGTTCAG

Supplementary Table 5. Sequences of the probes used in EMSA.

Probes	Sequences (5´-3´)
SP1 and ETS1-1-Biotin-labeled	F: GCGCCTTTCCCGAGCACGGGCGGGGAAAAG
	R: CTTTTCCCCGCCCGTGCTCGGGAAAGGCGC
SP1 and ETS1-1-Cold	F: GCGCCTTTCCCGAGCACGGGCGGGGAAAAG
	R: CTTTTCCCCGCCCGTGCTCGGGAAAGGCGC
SP1-Cold mutated	F: GCGCCTTTCCCGAGCACGttttGGGAAAAG
	R: CTTTTCCCAAAACGTGCTCGGGAAAGGCGC
ETS1-1-Cold mutated	F: GCGCCTTTaaCGAGCACGGGCGGGGAAAAG
	R: CTTTTCCCCGCCCGTGCTCGTTAAAGGCGC
ETS1-2-Biotin-labeled	F: GAGCGGAAGAACTTCCTGCAGCCAA
	R: TTGGCTGCAGGAAGTTCTTCCGCTC
ETS1-2-Cold	F: GAGCGGAAGAACTTCCTGCAGCCAA
	R: TTGGCTGCAGGAAGTTCTTCCGCTC
ETS1-2-Cold mutated	F: GAGCGGAAGAACTTaaTGCAGCCAA
	R: TTGGCTGCATTAAGTTCTTCCGCTC

Underlined sequences represent the binding sites and lower-case sequences represent the mutated sites.
